# Assessment health status of ICU medical equipment levels at Neyshabur hospitals using ICNA and ACC indices

**DOI:** 10.1016/j.mex.2018.10.016

**Published:** 2018-10-23

**Authors:** Hossein Najafi Saleh, Ali Kavosi, Manizhe Pakdel, Mahmood Yousefi, Farzaneh Baghal Asghari, Ali Akbar Mohammadi

**Affiliations:** a Department of Environmental Health Engineering School of Health, Torbat Heydariyeh University of Medical Sciences, Torbat Heydariyeh, Iran; b Nursing Research Center, Faculty Member, Golestan University of Medical Sciences, Gorgan, Iran; c Students Research Committee, Neyshabur University of Medical Sciences, Neyshabur, Iran; d Department of Environmental Health Engineering, School of Public Health, Tehran University of Medical Sciences, Tehran, Iran; e Department of Environmental Health Engineering, Neyshabur University of Medical Sciences, Neyshabur, Iran

**Keywords:** The study was performed in a ten-week period, twice a week before and after daily cleansing in contrary to the ICNA observational method and the ACC microbial method were performed on the selected sites, Intensive care units (ICU), Health status, ICNA, ACC, Hospitals, Disinfection

## Abstract

This study was conducted to evaluate the health status of medical equipment’s in Neyshabur hospital’s intensive care units (ICU) before and after daily cleaning in order to compare the efficiency of the observational and microbial methods in evaluating hygienic conditions and cleaning of the environmental surfaces at the hospitals in Neyshabur. The study was performed in a ten-week period, twice a week before and after daily cleaning according to the ICNA observational method and the ACC microbial method were performed on the selected sites. (before and after daily cleaning in order to compare ICNA observational method and the ACC microbial method which performed on the selected sites). Result showed in total, 826 ICNA checklists were completed in this research for the 13 studied spots, 27.12% of the spots were contaminated before cleaning procedures, which dropped to 7.75% after cleaning. Data of the samples using the ACC index revealed that 74.82 were contaminated and 7.75% were clean. Bottle suction with 8.2% and Electroshock with 1% were the most and the least contaminated spots, respectively. As the results proved, the microorganism of Staphylococcus epidermises is the most grown organism in the intensive care unit. This study suggests that visual assessment is not enough to ensure quality of the process and it is necessary to document the level of cleanliness by quantitative methods. Also preparing the integrated instructions and guidelines of cleaning and disinfection and its continuous monitoring with standard methods would be effective in reducing the microbial contamination.

## Graphical abstract

**Figure:**
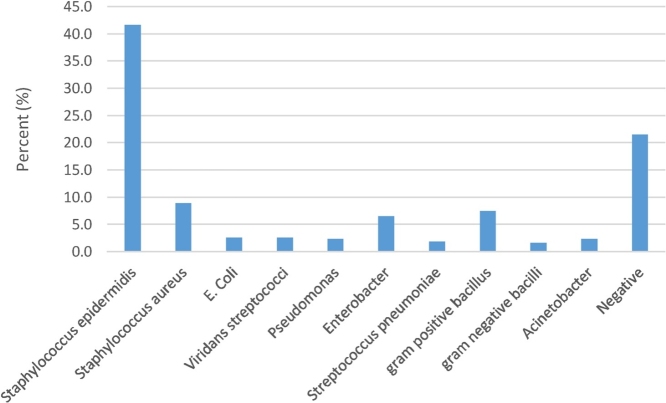

**Specifications Table**

**Table:** 

Subject area	Environmental Science; Hospital Environment
More specific subject area	Environmental cleaning
Method name	The study was performed in a ten-week period, twice a week before and after daily cleansing in contrary to the ICNA observational method and the ACC microbial method were performed on the selected sites This study was conducted to evaluate the health status evaluation of medical equipment’s in hospital’s intensive care units (ICU) before and after daily cleaning and to compare the efficiency of the observational and microbial methods in evaluating hygienic conditions and cleaning of the environmental surfaces at the hospitals.
Name and reference of original method	Sherlock O, O’Connell N, Creamer E, Humphreys H. Is it really clean? An evaluation of the efficacy of four methods for determining hospital cleanliness. Journal of Hospital Infection 2009:72; 140-146. Riyahin A A, and et al. Evaluation of ICU microbial contamination in Qom hospitals using observational and microbial monitoring methods with three indices of observation, colony count, and Methicillin-Resistant S. Aureus. Journal of current research in science, 2014, 2, No. 6, pp: 788-793.
Resource availability	The data are available with this article

## Method details

An important way to promote disease recovery is to clean the patient's environment. Due to the high volume of patients, the speed and severity of patient care activities by health care workers, irregularities in the levels of the hospital and medical equipment that require fluent cleaning, have made the hospital's health condition susceptible to possible pathogens. Recent studies on the quality of environmental cleanup in hospitals suggest that despite the efforts to clean up levels, most microbial contamination is at an existing level [1,2]. Potential pathogens that have long been present in inanimate surfaces has been reviewed previously. The life span of some organisms in the hospital environment varies from a few weeks to several months. It has been documented that patients who are at increased risk of acquiring a multidrug-resistant organism (MDRO) if the room in which it was hospitalized has been contaminated, instead of being cleaned every day, should be replaced [3,4]. During the last decade, the increasing number of cases of hospital infection has raised a lot of attention [5]. Estimates show that the rate of hospital infection in the United States is 1.7 million cases a year, causing 99,000 deaths [6]. The emergence of hospital infections such as other infections depends on three components of the infectious agent, the host and the environment. Investigating the prevalence and incidence of hospital infections confirmed the association between the poor condition of the hospital environment health and the transmission of microorganisms which cause hospital infections [7]. Transmission of microorganisms from the environmental levels to patients is largely carried out through contact with surfaces [8]. The environmental surfaces in hospitals and health centers are divided into two categories of medical equipment such as handles, dialysis machines, photographic apparatus, trail, medical and pharmaceutical instruments, dental units, and Pseudo-homemade environmental surfaces such as walls, floors, and so on [9]. Environmental contamination, such as bedside table, cabinets and handles that are adjacent to the patient, has been confirmed to be important pathogens for hospital infections that can survive long-term environmental exposure and transmit illness [10]. Medical equipment such as barometer cuff, Stethoscope, hemodialysis machine, and photographic apparatus may also infect infectious agents and facilitate the transmission of these factors, cause the spread and outbreak of the disease [11]. Colonization of points such as bed protection and guide rails (bedside), bedside table, ventilator surfaces, sinks, suction cups, anesthetic equipment, curtains, buckets and shelters, door handles, Stethoscope and incubators are approved for Acinetobacter [6]. Vancomycin-resistant Enterococci (VRE) transmissions have been directly done to infected medical equipment, such as Electro Cardiograph (ECG) leads and thermometer probes [12]. The role of medical devices and equipment in the hospital, including stretcher and rectal thermometers, has been proven in the transmission of Clostridium difficult to patients [13]. Epidemiological studies demonstrate that environmental surfaces can also contribute to the transmission of respiratory and gastrointestinal infections [17] hence; proper hospital care and sanitation are two of the most important components of hospital infection control policies. The hospital environment health includes a wide range of routine activities that are significant because of their importance in controlling and managing hospital infections. One of these activities is cleaning and disinfection [7]. Although the purpose of themis not to create a sterile environment, adequate care is needed to remove objects, dirt, garbage, germs and pathogens to minimizie the risk of transmission of infection from the hospital environment to patients [14]. On the other hand, the presence of sensitive hosts in health centers and hospitals has led to special attention to the importance of these environments in controlling hospital infections [15]. From this perspective, controlling and limiting the spread of pathogens associated with health centers over the past decade has been one of the main issues discussed in the epidemiology of health care centers [16]. In one study, the level of environmental contamination and medical equipment was investigated in the intensive care unit during the long-term outbreak of acinetobacter, and infection of acinetobacter, clostridium difficile and methicillin-resistant Staphylococcus aureus (MRSA) was observed in a number of touch points close to the patients so the importance of the relationship between the environmental infection rate and colonization of the patients were confirmed [5]. On the other hand, the results of the studies have confirmed that the intensification of cleaning and upgrading the level of health can reduce the incidence of hospital infections caused by clostridium difficile and acinetobacter [6]. Recently, many studies have shown that routine cleaning and decontamination of the environment, on average, reduces the risk of transmission of MRSA and VRE by about 40% [[17], [18], [19]]. From this perspective, the proper management of the cleaning process in the hospital is essential and the maintenance of cleanliness is one of the effective elements of cleaning management [20]. An effective and economical way to reduce the incidence of hospital infections is cleaning, the process of which must be scientifically evaluated taking into account output and measurable results. Standard methods for assessing the efficacy of surveillance are observational or ocular monitoring and microbial one. [21]. unfortunately, most of the strategies for assessing the quality of health and safety of hospitals have limited the use of observational instruments which the observational method cannot indicate a microbial hazard [22]. Previous studies also show that while after cleaning, 82% of the examined sites in the sectors were characterized by a clean and appropriate observational assessment method, only 30% of them had a clean and hygienic microbial index [23]. Performing microbial sampling of the surfaces can be effective in the effectiveness of the newly improved cleaning, disinfection and disinfection procedures. These samples can also be used in epidemiological studies, determining the role of environmental and medical equipment surfaces as a reservoir or source of transmission of hospital infections [15]. Today, in the diagnosis and treatment of the majority of patients, medical equipment is used. However, there are no guidelines for controlling the daily cleaning of hospital equipment, and there is no study on the level and role of medical equipment surfaces in incidence and prevalence of nosocomial infections. The ICU is one of the main departments of most hospitals; this section usually has the highest bed occupancy rate among hospital departments; Since patients who admitted to this unit often due to reasons such as a defect or weakness in the immune system, the length of hospitalization, the need for continuous monitoring of the vital condition and perform invasive procedures, etc. use medical equipment more than other ones in their treatment, so more than others are at risk of hospital infections. Therefore, this study was conducted to evaluate the environmental health status of medical equipment before and after cleaning and disinfection in the hospital, and the evaluation of the effectiveness of the cleaning process, as well as to assess the effectiveness of the Infection Control Nurses Association (ICNA) (Nurses' Infection Control Community Checklist For monitoring of each point) compared with aerobic colony count (ACC) method (culturing and counting grown aerobic colony) are evaluated in assessing the health and cleaning conditions of hospital equipment surfaces.

## Material and methods

A cross sectional study had been conducted in ICU unit in one of hospitals at Neyshabur city, Eastern of Iran, for the period from April 2015 to April 2016. A total of, 826 ICNA checklists were completed in this research for the 13 studied spots including Tralee drug, stethoscope, barometric cuffs, nursing office, nursing station, EKG machine, telephone, refrigerator, bag valve mask, suction, ventilator, pulse oximetry and defibrillation are monitored as follows. The study was performed in a ten-week period, twice a week before and after daily cleaning according to the ICNA observational method and the ACC microbial method were performed on the selected sites [24].

## Observation monitoring

This audit methodology is used to monitor the health status, cleaning the environmental levels, the levels of medical equipment, aids and the effectiveness of the cleaning process in the prevention and control of hospital infections in many countries including the United Kingdom.

In this method, the points are evaluated by a standard checklist with eye direct observation method and the results are reported as acceptable (clean and sanitary) and unacceptable (dirty and non-sanitary). In this study, using an inferential checklist from the ICNA standard checklist, the points that were free from dirt, stains, contamination and impaction of the objects, rust, rupture, fracture, failure or any unsatisfactory agent were revealed as clean and acceptable health assessment; And the points with each of these defects were considered dirty and unacceptable health [24].

## Microbial monitoring

First, before the sampling and testing, all of the used equipment was packed standard and sterilized using autoclave; for ensuring of sterilization, we used class 6 indicators in all packs and at the time of sampling, disposable sterile gloves were also used. After the sterile operation, sampling was performed using swabs of the selected locations and locations of medical equipment and medical aids. The sampling method was firstly wet the swab with sterile normal saline solution, then 10 cm^2^ of the selected spot level was swirled in zigzag, put the swab into a test tube containing 1 ml of sterile normal saline and ten seconds were mixed. After that, one hundred microliter of solution was cultured in two plates containing the Blad Agar and Muller Hinton medium prepared according to the pre-prepared order. Plates were incubated for 48 h at 37 °C and after this time, we calculated and recorded the growth of bacteria with a colony counter based on cfu/cm^2^; EMB medium was used to detect the type of grown microorganisms. According to previous studies, the points with microbial load greater than 2.5 cfu/cm^2^ were unacceptable (dirty and non-sanitary) and points with a microbial load of less than 2.5 cfu/cm^2^ were acceptable (clean and hygienic) [24].

## Result

**Fig. 1:**
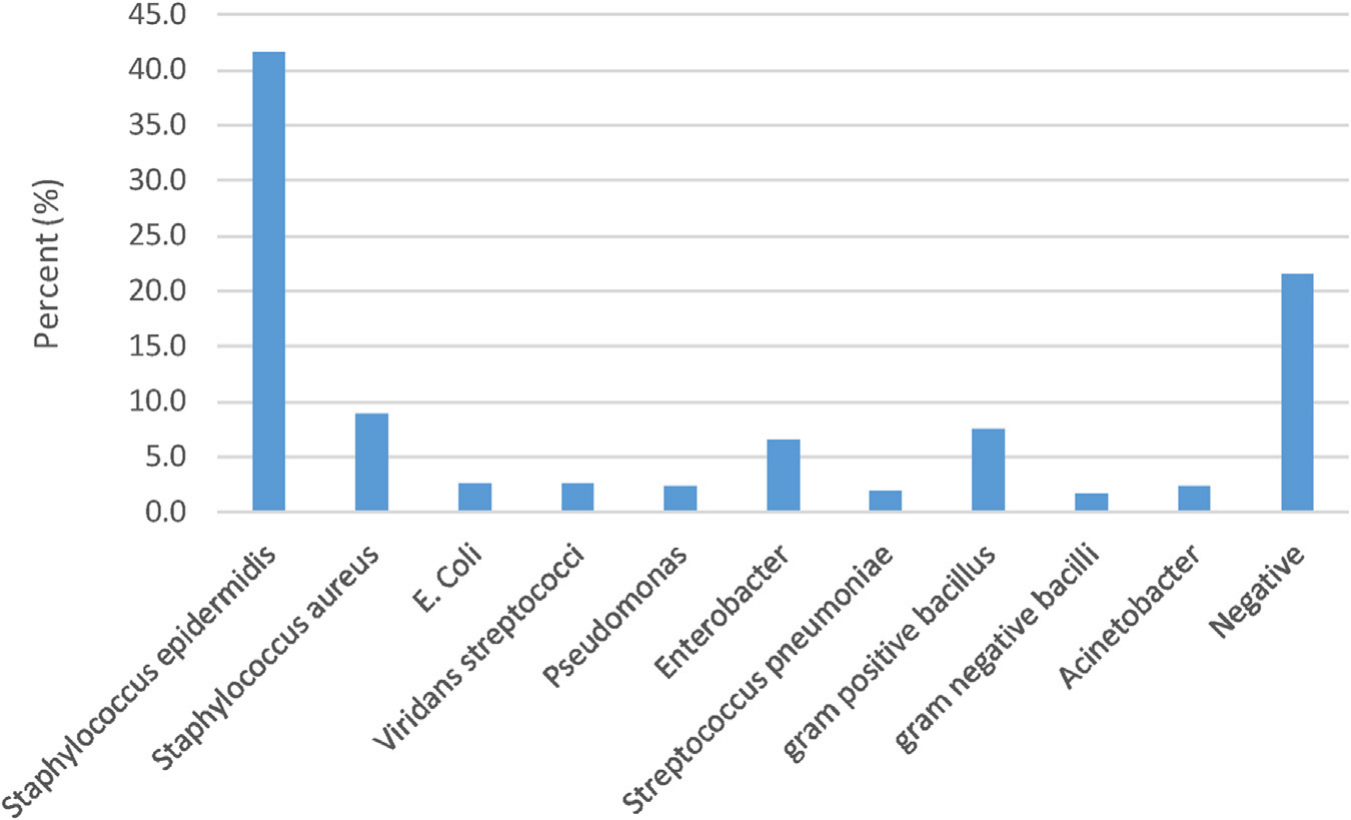
Microbial cultures of medical equipment of the intensive care unit prior cleaning (before disinfection).

**Fig. 2:**
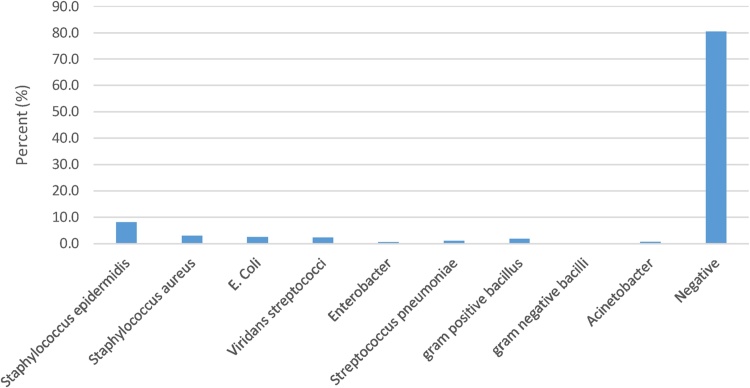
Microbial cultures of medical equipment of the intensive care unit after disinfection.

The results of using the standard checklist for monitoring clean or contaminated medical equipment in the intensive care unit of the hospital before and after using antiseptic are presented in Table 1. Also the results of the findings regarding the use of colony culture method to monitor the health of the medical equipment in the intensive care unit of the hospital before and after using antiseptic is accordance with Table 2.Result showed in total, 826 ICNA checklists were completed in this research for the 13 studied spots, 27.12% of the spots were contaminated before cleaning procedures, which dropped to 7.75% after cleaning. The results of microbial culture of the samples before and after the disinfection process are shown in Fig. 1, Fig. 2. As the results show, the microorganism of Staphylococcus epidermises is the most grown organism in the intensive care unit. The results of various medical equipment contamination based on the ICNA checklist before and after the disinfection process are described in Table 3, Table 4 respectively. Also the results of various medical equipment contamination based on the ACC index before and after the disinfection process are described in Table 5, Table 6.

**Table 1:** Monitoring the health status of medical equipment of the intensive care unit according to the checklist method.

Before using disinfectant		
	Number	Percent
Clean	602	72.9
Contaminated	224	27.1
Total	826	100
After using disinfectant		
Clean	762	92.3
Contaminated	64	7.7
Total	826	100

**Table 2:** Monitoring the health status of ICU medical equipment according to microbial culture method.

Before using disinfectant		
	Number	Percent
Clean	208	25.2
Contaminated	618	74.8
Total	826	100
After using disinfectant		
Clean	682	82.6
Contaminated	144	17.4
Total	826	100

**Table 3:** Frequency percentage of infected points based on the ICNA index prior disinfection.

Medical.equipments	ICNA.prior cleaning	
	Clean (%)	Contaminated (%)
Patient bedside table	6.3	1.45
Estethoscope	1.45	6.29
Blood pressure cuff	5.08	2.66
Nursing station	6.05	1.69
EKG	5.33	2.18
Medicine refrigerator handle	5.33	2.42
Bottle suction	2.18	6.54
Ventilator	6.78	1
Electroshock	6.78	1
Telephone receiver	7.26	0.48
Pulse oximetry	7.02	0.48
Patient bed	7.02	0.24
Ambu bag	6.29	0.73
Total	72.88	27.12

**Table 4:** Frequency percentage of infected points based on the ICNA index t after disinfection.

Medical.equipments	ICNA prior cleaning	
	Clean (%)	Contaminated (%)
Patient bedside table	7.75	0.00
Estethoscope	6.29	1.45
Blood pressure cuff	5.57	2.18
Nursing station	7.75	0.00
EKG	7.02	0.48
Medicine refrigerator handle	7.75	0.00
Bottle suction	5.33	3.39
Ventilator	7.75	0.00
Electroshock	7.75	0.00
Telephone receiver	7.75	0.00
Pulse oximetry	7.51	0.00
Patient bed	7.26	0.00
Ambu bag	6.78	0.24
Total	92.25	7.75

**Table 5:** Frequency of contaminated points based on the ACC index prior disinfection.

Medical.equipments	ACC prior cleaning	
	Clean (%)	Contaminated (%)
Patient bedside table	2.18	5.57
Estethoscope	1	6.78
Blood pressure cuff	0.24	7.51
Nursing station	0.73	7.02
EKG	5.33	2.18
Medicine refrigerator handle	0	7.75
Bottle suction	0.48	8.23
Ventilator	5.08	2.66
Electroshock	6.78	1
Telephone receiver	0.48	7.26
Pulse oximetry	0.73	6.78
Patient bed	1.42	5.81
Ambu bag	0.73	6.29
Total	25.18	74.82

**Table 6:** Frequency of contaminated points based on ACC index after cleaning (disinfection).

Medical.equipments	ACC after cleaning	
	Clean (%)	Contaminated (%)
Patient bedside table	6.29	1.45
Stethoscope	6.29	1.45
Blood pressure cuff	4.36	3.39
Nursing station	7.02	0.73
EKG	6.29	1.21
Medicine refrigerator handle	7.51	0.24
Bottle suction	6.78	1.94
Ventilator	6.78	0.98
Electroshock	6.78	0.98
Telephone receiver	7.75	0.00
Pulse oximetry	5.57	1.94
Patient bed	5.33	1.94
Ambu bag	5.81	1.21
Total	82.57	7.75

## Discussion

In total, 826 ICNA checklists were completed in this research for the 13 studied spots. Altogether, 27.12% of the spots were contaminated before cleaning procedures, which dropped to 7.75% after cleaning. Data of the samples using the ACC index revealed that 74.82were contaminated and 7.75% were clean. Bottle suction with 8.2% and Electroshock with 1% were the most and the least contaminated spots, respectively. As the results reported, the microorganism of Staphylococcus epidermises is the most grown organism in the intensive care unit. In a study by Sherlock et al. in a 700-bed hospital in Ireland in 2009, the hospital's health and well-being process was assessed using the ICNA visual index and the ACC microbial index in both internal and surgical sections. Results showed that ICNA using an observation checklist to monitor any point (5.5%) and using an ACC microbial method (sampling with swab, culture, and counting of colonies grown), 7.9% of the sites were unhealthy [14]. In another study by Alhammad et al. in Manchester, England 2008, during a period of 4 weeks in intensive care units in hospital, a general average of total microbial load before cleaning was 2.89 ± 0.89 and after that 1.05 ± 0.18 reported. These numbers were reported to be 3.45 ± 0.69 for locations that were not cleaned regularly. In total of 116 locations, 9 of them (7.7%) were found to have a higher microbial load from 5 cfu / cm^2^ [15]. In 2009, Anderson et al. evaluated the effects of bottom floor cleaning with four dry, spray, lime and wet drying procedures in 4 rooms of a hospital. They were screened before and after cleaning with each sampling method for determining the microbial load of the floor and the average microbial load before cleaning was 83.20. The mean removal rate was 60% in dry, wet and soaked methods, and in the spray method only 30% of the initial microbial load was obtained [25]. Griffith et al. conducted a study, in order to evaluate routine cleaning efficacy and to compare the effects of changing the cleaning program using antiseptic and detergents. The results of the observational assessment of most surfaces, except for the toilet valve and the phone were clean, while in the ACC microbial monitoring method, six points out of nine had an infection higher than the standard level, with the toilet tap for patients with an average ACC of 9.5 had the highest contamination, which resulted in modification of the method and processing to an average of less than one acceptable level. The average amount of ACC microbial load in all sampling points was found to be significant after the correction and change of method [26]. In a study by Whit et al. in 2008, 10 points of ICU surfaces in terms of health and wellness were surveyed, out of a total of 200 samples taken during the study, about 25% of them were unacceptable. Most of the non-health samples were related to the surfaces of medical equipment, beds and curtains [21]. Malik and Cooper conducted a study (2003) in two parts of the internal and surgical section of four hospitals in England immediately after routine cleaning of selected sites with an ocular and microbial observation index. They reported that ninety percent of the points in the surgical wards and 100% of the points in the internal ward were evaluated with an acceptable observational index, while the microbial susceptibility in the surgery section was only 10% of the points in terms of acceptable cleanliness [20]. Cooper et al. assessed a 27-point study in two parts of the internal and surgical section in England and Wales before and after the cleaning with observation and microbial load methods. The mean differences between points with an unacceptable health status with the observational and Microbial were reported 65.75. Overall, 89.28% of the points with ocular index and 24.6% were declared acceptable by microbial index [27]. The study of Hatami et al. in 2016 in Hospitals in Kermanshah, Iran, showed that the Staphylococcus saprophyticus (74.5%), Bacillus, Coryneabacterium, were the predominant isolates among Gram positive and Enterobacter spp, E Coli and Klebsiella were the predominant isolates among negative bacteria, respectivelyr [28]. In a research conducted by Riahi et al. in Qom in 2014, the health status of environmental surfaces in the intensive care unit before and after daily cleaning was observed by observational and microbial culture method. In this study, 9 points that were more likely to be contaminated were selected for sampling and monitored for 10 weeks. The results showed that in the observational method, the percentage of contaminated points before and after cleaning was 59% and 41.5%, respectively. In the microbial method, these percentages were 57.5% and 47.5% [24].

## Conclusion

Hospital infection are one of the most important infectious diseases. The relationship between undesirable hygienic conditions in the environment of the hospitals and the transmission of microorganisms has been confirmed in various studies. Results of this study showed that the frequency of contaminated spots determined by using all three indicators of observation, colony count, and S. aureus declined after cleaning in comparison with before cleaning. Also Results of this study show that cleaning can be an effective method in reducing microbial loads in the environment. One of the limitations of this study was the selection of sampling sites that were selected for removal of this limitation of sampling in most of the locations. Another limitation is that only one type of disinfectant was used to evaluate the efficiency. Therefore, it is better to use different disinfectants in future studies.

## Conflict of interest

The authors of this article declare that they have no conflict of interests.
